# The OPTIMIST-A trial: evaluation of minimally-invasive surfactant therapy in preterm infants 25–28 weeks gestation

**DOI:** 10.1186/1471-2431-14-213

**Published:** 2014-08-27

**Authors:** Peter A Dargaville, Camille Omar F Kamlin, Antonio G De Paoli, John B Carlin, Francesca Orsini, Roger F Soll, Peter G Davis

**Affiliations:** 1 Department of Paediatrics, Royal Hobart Hospital and University of Tasmania, Liverpool Street, Hobart TAS 7000, Australia; 2 Menzies Research Institute Tasmania, Hobart, Australia; 3 Neonatal Services, Royal Women’s Hospital, Melbourne, Australia; 4 Department of Obstetrics and Gynaecology, University of Melbourne, Melbourne, Australia; 5 Murdoch Childrens Research Institute, Melbourne, Australia; 6 Clinical Epidemiology and Biostatistics Unit, Murdoch Childrens Research Institute, Melbourne, Australia; 7 Department of Paediatrics, University of Melbourne, Melbourne, Australia; 8 Department of Neonatology, Fletcher Allen Health Care; University of Vermont, Burlington, Vermont, USA

**Keywords:** Infant, Preterm, Respiratory distress syndrome, Continuous positive airway pressure, Pulmonary surfactants, Bronchopulmonary dysplasia

## Abstract

**Background:**

It is now recognized that preterm infants ≤28 weeks gestation can be effectively supported from the outset with nasal continuous positive airway pressure. However, this form of respiratory therapy may fail to adequately support those infants with significant surfactant deficiency, with the result that intubation and delayed surfactant therapy are then required. Infants following this path are known to have a higher risk of adverse outcomes, including death, bronchopulmonary dysplasia and other morbidities. In an effort to circumvent this problem, techniques of minimally-invasive surfactant therapy have been developed, in which exogenous surfactant is administered to a spontaneously breathing infant who can then remain on continuous positive airway pressure. A method of surfactant delivery using a semi-rigid surfactant instillation catheter briefly passed into the trachea (the “Hobart method”) has been shown to be feasible and potentially effective, and now requires evaluation in a randomised controlled trial.

**Methods/design:**

This is a multicentre, randomised, masked, controlled trial in preterm infants 25–28 weeks gestation. Infants are eligible if managed on continuous positive airway pressure without prior intubation, and requiring FiO_2_ ≥ 0.30 at an age ≤6 hours. Randomisation will be to receive exogenous surfactant (200 mg/kg poractant alfa) via the Hobart method, or sham treatment. Infants in both groups will thereafter remain on continuous positive airway pressure unless intubation criteria are reached (FiO_2_ ≥ 0.45, unremitting apnoea or persistent acidosis). Primary outcome is the composite of death or physiological bronchopulmonary dysplasia, with secondary outcomes including incidence of death; major neonatal morbidities; durations of all modes of respiratory support and hospitalisation; safety of the Hobart method; and outcome at 2 years. A total of 606 infants will be enrolled. The trial will be conducted in >30 centres worldwide, and is expected to be completed by end-2017.

**Discussion:**

Minimally-invasive surfactant therapy has the potential to ease the burden of respiratory morbidity in preterm infants. The trial will provide definitive evidence on the effectiveness of this approach in the care of preterm infants born at 25–28 weeks gestation.

**Trial registration:**

Australia and New Zealand Clinical Trial Registry: ACTRN12611000916943; ClinicalTrials.gov: NCT02140580.

## Background

### The CPAP-surfactant dilemma

In the past two decades, exogenous surfactant therapy has been a cornerstone of therapy for preterm infants, and is known to be effective when given prophylactically in the delivery room, or as a rescue therapy to infants with established respiratory distress syndrome (RDS) [1]. Its introduction into neonatal practice in the early 1990s was followed by a considerable decrease in overall neonatal mortality rate [2]. With the evolution and refinement of intensive care for preterm infants, the place of exogenous surfactant therapy is changing. The more widespread use of nasal continuous positive airway pressure (CPAP) as a primary means of respiratory support means many preterm infants with respiratory distress now avoid intubation in the delivery room or in early post-natal life [3-6]. This approach also means delaying or avoiding administration of surfactant.

In preterm infants ≤29 weeks gestation, the potential advantages of early CPAP have been highlighted in large randomised controlled trials in which treatment with CPAP from birth, without administration of surfactant, resulted in fewer ventilator days [7-9] and a trend towards lower risk of bronchopulmonary dysplasia (BPD) compared to intubated controls [7-9]. In these trials, however, a large number of infants starting on CPAP ultimately required intubation at some time. In the COIN trial [7], 46% of infants who commenced on CPAP went on to be intubated in the first 5 days (at a median age of 6.6 hrs), with increasing oxygen requirement and/or respiratory acidosis being the most prominent reasons for intubation. A further 13% of CPAP-treated infants required intubation beyond 5 days. In the SUPPORT study [8] more than 75% of infants randomised to the CPAP group were intubated at some time, and 67% received surfactant. In the VON study [9], 52% of infants commencing on CPAP without prior surfactant therapy ultimately required intubation, and 44% received surfactant. These findings appear to confirm those of earlier observational studies demonstrating that the most usual cause of early CPAP failure in preterm infants is unremitting RDS [5,6]. Widespread application of CPAP for initial respiratory support in preterm infants provides benefit for many, but is to the detriment of a significant minority of infants destined to go on to fail CPAP because of surfactant deficiency.

### CPAP failure and adverse outcome

The group of preterm infants failing CPAP has been incompletely characterised to date. Our research team has therefore examined the respiratory course and outcome for a large cohort of preterm infants initially managed on CPAP at Royal Hobart Hospital, Hobart (RHH) and Royal Women’s Hospital, Melbourne (RWH) [10]. We found that CPAP failure, defined as need for intubation before 72 hrs, was associated with a high risk of adverse outcome. Infants who failed CPAP and were intubated < 72 h had a substantially longer duration of respiratory support than those in whom CPAP was successful. At 25–28 weeks, infants failing CPAP had a higher risk of mortality, BPD, death or BPD, and necrotising enterocolitis (NEC) [10].

As noted by other investigators [6,7], CPAP failure in the RHH-RWH preterm cohort most often occurred in the context of unremitting RDS, with the median FiO_2_ at intubation being 0.50 in the 25–28 week infants failing CPAP, and 0.44 in the 29–32 week group [10]. In 23% of cases a pneumothorax was present at the time of intubation. Hypoventilation (PCO_2_ > 60 mmHg) was a contributing factor in only 15% of cases overall [10].

### Intubation for surfactant administration

Given the above, it is conceivable that outcomes for preterm infants managed initially with CPAP could be further improved if the subgroup of infants showing signs of surfactant deficiency were to receive exogenous surfactant. Recognizing the merits of surfactant, especially when given early [1,11,12], some clinicians choose to intubate infants on CPAP solely for the purpose of administering surfactant, followed by immediate extubation and return to CPAP (the “INSURE” approach – ***in***tubation, ***sur***factant, ***e***xtubation) [13-15]. Several clinical trials of this technique have pointed to reductions in the need for subsequent mechanical ventilation and further surfactant therapy [16-20], and the risk of pneumothorax [20]. A more recent study in infants 25–28 weeks gestation did not find a difference in the primary outcome of need for mechanical ventilation during the first 5 days, but 10% of those intubated solely for surfactant administration could not be extubated within 1 hour and were thus deemed to have reached the primary outcome [21]. A larger proportion (17%) were not able to be extubated in the INSURE group in the recent Vermont-Oxford Network trial [9].

Intubation solely for administration of surfactant is a common practice in many Scandinavian units [3,22], but is less popular elsewhere. Many clinicians consider the potential benefits of surfactant are outweighed by the risks of intubation. In the delivery room, intubation can be complicated by multiple intubation attempts and episodes of hypoxia [23]. Beyond the delivery room, intubation in preterm infants is now rarely performed without pre-medicating with narcotics ± muscle relaxants [24], meaning that there may be a delay in extubation once surfactant has been administered. Such a delay has been observed in at least one clinical trial of intubation for surfactant therapy in infants on CPAP [21]. The use of sedating premedication also means that where surfactant is given immediately after intubation, as it most usually is, the consequent suppression of respiratory effort may impair surfactant distribution. Experimental data suggest that surfactant administration in a spontaneously breathing subject results in more effective dispersion and greater tissue incorporation of phospholipid [25].

### Minimally-invasive surfactant therapy

In view of the difficulties associated with intubation for surfactant delivery, less invasive means of delivering surfactant have been pursued. Several techniques of “minimally-invasive surfactant therapy” (MIST) have been described in which surfactant is delivered without tracheal intubation, including nasopharyngeal instillation [26], laryngeal mask placement [27] and aerosolisation [28]. None of these methods appears ready for clinical application on a wider scale at present. Another method of MIST in which the trachea is catheterised with a feeding tube has been reported [29-32]. The technique involves insertion of a 5 French gauge feeding tube into the trachea with Magill’s forceps. Surfactant is then administered over 1–5 minutes, and the catheter thereafter removed. A randomised controlled trial of MIST using this technique (the AMV trial) has recently been conducted in infants 26–28 weeks gestation having FiO_2_ > 0.30 in the first 12 hours [33]. Compared to controls, surfactant-treated infants had a lower rate of subsequent mechanical ventilation (28% vs 45%); no difference in the rate of pneumothorax or other adverse events was noted. A further trial comparing this method of MIST with standard intubation in very preterm infants (23–26 weeks gestation) has now been completed, and the results are awaited.

An alternative approach in which a flexible feeding tube is passed through the vocal cords without using Magill’s forceps has recently been reported [34]. Surfactant delivery with this method was compared with INSURE in infants <34 weeks gestation, with the finding of a reduction in early mechanical ventilation, and a decreased incidence of BPD. This method would amount to a procedural challenge for most practitioners, and is thus unlikely to be widely adopted.

### The “Hobart method” of MIST

Surfactant instillation by flexible feeding tube has several technical difficulties that may limit its widespread application. Clinicians who solely practice oral intubation will be unfamiliar with Magill’s forceps, and may find them cumbersome and hard to use. Additionally, the highly flexible feeding tube may on occasions be difficult to insert through the vocal cords, and also difficult to maintain in position once inserted. For these reasons, and with the recognition of the potential benefits of MIST, our research group has developed an alternative and novel MIST technique using a narrow bore vascular catheter (16 gauge Angiocath, Product No. 382259, Becton Dickinson, Sandy, UT, USA) [35]. This catheter has an external diameter of 1.7 mm, and a length of 135 mm, and is made from fluorinated ethylene propylene polymer. It has the dual properties of sufficient stiffness to allow guidance towards and beyond the vocal cords, and sufficient elasticity and softness to avoid damage to the vocal cords and other vital structures. This catheter can be advanced through the vocal cords under direct vision using a laryngoscope, without the need for Magill’s forceps. A curvature in the catheter can be fashioned if desired to facilitate placement. Surfactant can then be administered in one or several boluses, and respiratory support continued with nasal CPAP. A video of the technique can be accessed at the OPTIMIST-A trial website (http://www.menzies.utas.edu.au/optimist-trials).

### Clinical experience with the Hobart method

A preliminary evaluation of the Hobart method of MIST was conducted at RHH [35], and a two-site feasibility study was undertaken at RHH and RWH [36]. In the initial study at RHH, MIST was performed in 25 infants, of gestational age range 25–34 weeks and birth weight range 500–3000 g [35]. The MIST procedure was performed in the delivery room in 2 cases, and after arrival in the Neonatal Intensive Care Unit (NICU) in 23. No pre-medication was used. Surfactant (Curosurf, Chiesi Farmaceutici, Parma, Italy) was delivered at a dosage of approximately 100 mg/kg, given in 1 or 2 boluses. The surfactant was successfully administered in every infant, with two attempts at catheterisation needed in 9 (35%). Brief bradycardia (heart rate <100 beats per minute) was noted in 11 infants (44%), usually contemporaneous with insertion of the laryngoscope blade, and in all cases self-resolving within 10 seconds. Positive pressure inflations were required after surfactant administration in 11 infants (44%).

The further feasibility study of the Hobart method of MIST enrolled 61 infants of 25–32 weeks gestation [36]. Eligibility for MIST was based on the need for CPAP pressure ≥7 cm H_2_O and FiO_2_ ≥ 0.30 (25–28 weeks) or ≥0.35 (29–32 weeks). At RHH, 3 infants in the 25–28 week gestation group were treated with FiO_2_ < 0.30; each had a CPAP pressure of 8 cm H_2_O and signs of respiratory distress. Overall, the 25–28 week group received MIST at a mean age of 3.5 ± 3.5 hrs (mean ± SD), and the 29–32 week infants at 10.8 ± 7.5 hrs. Surfactant was successfully administered in all cases, with two catheterisation attempts required in 20%. Positive pressure inflations by mask were used in 39% of infants prior to reinstitution of CPAP.

Respiratory course and outcomes in infants treated with MIST have been compared with like-gestation historical controls achieving the same CPAP and FiO_2_ thresholds (data from the RHH-RWH preterm CPAP cohort). Within each gestation range, the control infants were comparable to those treated with MIST in terms of median gestation, birth weight, exposure to antenatal corticosteroids, mode of delivery and Apgar score at 5 minutes. Several potential benefits of MIST were identified. FiO_2_ was more rapidly weaned in surfactant-treated infants than controls in the first 72 hrs. Need for intubation <72 hrs was diminished after MIST, most notably for infants at 25–28 weeks gestation (OR 0.21, 95% CI 0.083-0.55), but with a strong trend in the same direction in the 29–32 week group (odds ratio 0.34, 95% CI 0.11-1.06). Duration of oxygen therapy was reduced in infants treated with MIST at all gestations.

### The need for further randomised controlled trials of MIST

The findings of the evaluation of MIST using the Hobart method, coupled with the clear evidence that CPAP failure occurs largely because of unremitting RDS and is associated with adverse outcomes, have been the genesis of the OPTIMIST-A trial^a^. There is considerable scientific justification for this trial, with strong data in support of: a) the poor outcome for those failing CPAP, b) the capacity to identify such infants early, c) the potential for MIST to alter the outcome in such infants, and d) the potential benefits of surfactant delivery in the spontaneously breathing infant [25]. It is thus appropriate to subject MIST to the highest level of scientific scrutiny in the form of a randomised controlled trial.

### Important considerations in trial protocol development

#### Enrolment criteria

Not all preterm infants 25–28 weeks gestation managed on CPAP from the outset stand to benefit from surfactant administration with a minimally-invasive technique. Some have minimal or mild RDS, and are well supported by CPAP alone. For MIST to be of value, it must be coupled with early and accurate selection of infants at greatest risk of failing CPAP. In this regard, several indicators previously put forward have been rejected: a) radiological scores [6], which are confounded by variability of X-ray technique and subjectivity of interpretation, b) functional surfactant assays [37,38], which require specialised equipment and training, c) indices of oxygenation based on arterial pO_2_[6], which are impractical because so few infants on CPAP have arterial lines *in situ*, and d) Silverman clinical scores, which will vary considerably depending on the CPAP pressure level.

Using data from the RHH-RWH preterm CPAP cohort, we sought a bedside predictor of early CPAP failure in the early post-natal period in infants 25–28 weeks gestation. In a logistic regression model incorporating demographic variables, FiO_2_ and CPAP pressures, by far the strongest predictor of later need for intubation was the highest FiO_2_ in the first 2 hours. Addition of CPAP pressure improved the goodness of fit only slightly (R^2^ 0.5 vs 0.45). A similar regression analysis by De Jaegere et al. in infants <30 weeks gestation found FiO_2_ by 2 hours to be the most influential variable in prediction of later intubation [39]. Area under the receiver operating characteristic curve for prediction of CPAP failure using FiO_2_ was 0.83 in the RHH-RWH preterm CPAP cohort and 0.84 in the study of De Jaegere et al. [39]. On this basis, and in recognition of the need for simplicity in framing the entry criteria, highest appropriate FiO_2_ has been chosen as an entry criterion for the OPTIMIST-A trial. The FiO_2_ threshold of ≥0.30 is the same as that used in the AMV trial, and in our preterm CPAP cohort predicted intubation <72 hrs with a sensitivity of 83% and positive predictive value of 60%. Infants achieving this threshold in the first 2 hrs had a relatively high likelihood of later intubation (OR 5.6, 95% CI 1.7-18).

#### Surfactant dosage

Standard surfactant dosage for preterm infants with RDS ranges from 100 to 200 mg/kg. At least when using Curosurf, there is some evidence that a dose of 200 mg/kg reduces the need for re-treatment [40]. This dose has been used in several studies of surfactant administration by brief intubation in infants <30 weeks gestation [17,21]. In the feasibility studies of the Hobart method of MIST, 7 infants 25–28 weeks gestation received a surfactant dosage of 200 mg/kg. No treatment complications were noted in those receiving the larger dose, their oxygenation response was more pronounced and prolonged, and none required intubation <72 hrs [36]. These observations, coupled with the wider experience, provide the basis for the 200 mg/kg surfactant dosage stipulated in the OPTIMIST-A trial.

#### The surfactant delivery catheter

The surfactant delivery catheter used to date in the studies of the Hobart MIST method has been the 16G Angiocath (Becton Dickinson, Sandy, UT, USA). These studies revealed no major deficiencies with the catheter for the purpose of surfactant instillation, other than the need to mark the depth of insertion with a marker pen. The 16G Angiocath will thus be used in the OPTIMIST-A trial until a purpose-built surfactant delivery catheter with very similar design features becomes available.

#### Method of laryngoscopy

In the initial feasibility studies at RHH and RWH, direct laryngoscopy for tracheal catheterisation has been performed using a standard laryngoscope with a Miller 0 or 00 blade. During further evaluation, tracheal catheterisation has been successfully undertaken using a Glidescope Cobalt AVL video laryngoscope (Verathon Medical, Burnaby, Canada) with a size 0 blade. The use of this video laryngoscope is permitted in the OPTIMIST-A trial, and modifications of the device are being pursued to assist in guiding the catheter through the vocal cords.

#### Premedication

Experience from the RHH-RWH feasibility studies suggests that the MIST procedure is generally well-tolerated without any premedication. Initial evaluation of the Cologne method reported the use of atropine at a dose of 25 μg/kg [30], although this has since become optional [32]. In the OPTIMIST-A trial premedication with atropine is at the discretion of the OPTIMIST Treatment Teams. Use of oral sucrose is encouraged. Narcotic analgesics or other sedating medications are not permitted.

#### Intubation criteria

Criteria for intubation of infants on CPAP have been stipulated in the OPTIMIST-A trial, based on experience from previous studies, examination of the RHH-RWH data, and knowledge of local practices. Enrolled infants will be intubated if persistently requiring an FiO_2_ of 0.45. As with previous trials [7,8], other intubation criteria apply in the event of apnoea, persistent acidosis or need for an intervention.

#### Primary outcome and sample size

An initial randomised controlled trial of MIST had as its primary outcome the need for intubation and mechanical ventilation, and found a reduction in this outcome after MIST [33]. Whilst avoidance of mechanical ventilation is a worthy goal, it would seem that if infants are to undergo direct laryngoscopy and tracheal surfactant administration, it should be with the aim of producing a more tangible benefit than simply reducing the intubation rate. The choice of primary outcome in the OPTIMIST-A trial reflects this. The primary outcome is the composite of death or BPD, which according to our experience in 25–28 week infants (RHH-RWH data) currently occurs in 53% of those failing CPAP and 38% of those reaching the enrolment threshold. The OPTIMIST A trial has been powered to detect a reduction by one-third in this outcome (from 38% to 25%). Such a reduction appears to be a realistic target given that the rate of death or BPD in those succeeding on CPAP (not intubated in the first 72 hours) is 14%. It would also represent a major improvement in outcome for the 25–28 week group overall.

With the publication of feasibility studies and small clinical trials, there is a possibility that surfactant administration via MIST could become popular in the neonatal community before being adequately scrutinised. The OPTIMIST-A trial offers timely and rigorous evaluation of MIST, and we believe it will be a definitive trial in shaping the future approach to this therapy. For this reason we have calculated the sample sizes for the OPTIMIST-A trial based on 90% power.

### Trial aim

To evaluate in a randomised controlled trial the efficacy of surfactant delivery via a minimally-invasive technique in preterm infants 25–28 weeks gestation with RDS treated with CPAP.

### Trial hypothesis

That early surfactant administration via a minimally-invasive technique to preterm infants on CPAP results in a lesser duration of mechanical respiratory support, and a higher incidence of survival without BPD.

## Methods/Design

### Trial design

Multicentre, randomised, masked, parallel controlled trial.

### Participating centres

The following centres are actively recruiting for the trial:

**Australia**: Royal Hobart Hospital, Hobart; Royal Women’s Hospital, Melbourne; Monash Medical Centre, Melbourne; Women’s and Children’s Hospital Adelaide.

**New Zealand**: Auckland City Hospital, Auckland; Middlemore Hospital, Auckland.

**United States**: Evanston Hospital, Evanston, IL.

**Turkey**: Zekai Tahir Burak Hospital, Ankara.

The following centres have committed to joining the trial:

**Australia**: Mercy Hospital for Women, Melbourne.

**New Zealand**: Dunedin Hospital, Dunedin.

**Israel**: Ziv Medical Center, Tsfat; Bnai Zion Medical Center, Haifa.

**United Kingdom**: Southampton University Hospital, Southampton; Southern General Hospital, Glasgow; Royal United Hospital, Bath; University Hospital of Wales, Cardiff.

**The Netherlands**: University Medical Center, Groningen.

**Poland**: Poznań University of Medical Sciences, Poznań; Medical University of Łódź, Łódź;

Polish Mothers’ Memorial Hospital, Łódź; SPZOZ Provincial Hospital, Bydgoszcz.

**Italy**: San Gerardo Hospital, Monza; Ospedale Maggiore Policlinico, Milano.

**Slovenia**: University Medical Centre, Ljubljana.

**Greece**: Aristotle University of Thessaloniki, Thessaloniki.

**Turkey**: Uludag University Hospital, Bursa.

**United States**: Yale-New Haven Children’s Hospital, New Haven, CT; Children’s Hospital of Georgia, Augusta, GA; Cooper University Hospital, Camden, NJ; Kapiolani Medical Center, Honolulu, HI; Fletcher Allen Health Care, Burlington, VT; Beth Israel Deaconess Medical Centre, Boston, MA; West Virginia Health Science Center, Morgantown, WV; University of Southern California, Los Angeles, CA; Oklahoma University Health Science Center, Oklahoma, OK; Westchester Medical Center, Valhalla, NY.

### Study population

Preterm infants of gestation 25 weeks 0 days to 28 weeks 6 days who are inborn and admitted to the NICU of a participating study centre, and who fulfil the entry criteria detailed below.

### Recruitment

#### Entry criteria

1. Requiring CPAP or nasal intermittent positive pressure ventilation (NIPPV) because of respiratory distress.

2. CPAP pressure of 5–8 cm H_2_O and FiO_2_ ≥ 0.30.

3. Less than 6 hours of age.

4. Agreement of the Treating Physician in charge of the infant’s care.

5. Signed parental consent.

#### Exclusion criteria

1. Previously intubated, or in imminent need of intubation because of respiratory distress, apnoea or persistent acidosis.

2. Congenital anomaly or condition that might adversely affect breathing.

3. Identifiable alternative cause for respiratory distress (e.g. congenital pneumonia or pulmonary hypoplasia).

4. Lack of availability of an OPTIMIST treatment team.

### Consent

Written parental consent must be obtained prior to randomisation by the treating clinicians. A plain language document outlining the rationale for the study is given to the parents. Consent should be obtained prenatally where possible, in which case the infant will only be enrolled after birth if all inclusion and no exclusion criteria were fulfilled. In all cases, written consent is obtained using a specifically-designed consent form.

### Randomisation

Once consent has been obtained and all entry criteria are met with no exclusions, the infant is randomised by the OPTIMIST Treatment Team, after handover of care from the treating clinicians. Enrolled infants are randomised into “surfactant via MIST” and “standard care” (sham treatment) groups, with an allocation ratio of 1:1, using a web-based randomisation procedure that requires confirmation of eligibility criteria and consent before revealing the randomly determined allocation. The randomisation is in randomly permuted blocks of variable length, stratified by study centre, and by gestational age. For the OPTIMIST-A trial there are two gestational age strata (25–26 weeks and 27–28 weeks). Twins and higher order multiples are randomised independently. Infants who are unstable and in need of immediate intubation should not be randomised, even if consent has been obtained; such infants will not be considered to have been enrolled.

### Masking

In order to mask the group allocation from the treating clinicians, an OPTIMIST Treatment Team is mobilised to perform the randomisation and intervention. This team consists of a neonatologist, senior neonatal trainee or neonatal nurse practitioner, and a neonatal nurse, none of whom are currently involved in the infant’s care. Their role is to obtain the randomisation, and then within 1 hour, after screening the infant as effectively as possible from the treating clinicians, to administer the intervention (surfactant via MIST or sham treatment) in accordance with the randomised allocation. Their activities, including removal of surfactant from the medication refrigerator, movement and speech within the screened space, and manipulation of the infant, should be such that the treating clinicians cannot discern which intervention is received. All treating clinicians are made aware that the OPTIMIST Treatment Team will be concealing treatment allocation by performing a sham procedure on those infants randomised to standard care. The time taken to perform the intervention should be the same regardless of treatment allocation, and the infant is returned to the pre-intervention CPAP settings prior to removing the screens. A survey of clinical staff is being conducted after each OPTIMIST intervention in order to assess the success of masking.

Members of OPTIMIST Treatment Teams at all institutions undertake not to reveal the allocation group of randomised infants.

### Intervention

The intervention is performed in the NICU of participating centres. Prior to intervention, all neonates must be stable on CPAP delivered by prongs or mask. An intravenous cannula should be *in situ*. It is desirable that a blood gas analysis (arterial or capillary) is performed before intervention, although this is not mandatory. A chest X-ray is recommended to confirm the diagnosis of RDS, and to exclude other causes of respiratory distress.

Having been briefed on the current condition of the infant, the OPTIMIST Treatment Team screens the infant from treating clinicians as completely as possible. The infant is then randomised, and the allocated intervention carried out as soon as possible (maximum 1 h after randomisation). Pre-intervention observations are recorded. The Treatment Team takes a labelled box containing full or empty surfactant vials from the OPTIMIST canister in the medication refrigerator. This canister must not be accessed by any other person other than the NICU pharmacist responsible for replenishing the stock of surfactant, which is supplied specifically for the study.

### Intervention – surfactant administration via MIST

The following protocol is used for performing MIST:

#### Preparation

1. Prepare the 16G Angiocath by marking a point indicating the desired depth of insertion beyond the vocal cords with a marker pen. The required depth is as follows: 25–26 weeks: 1.5 cm; 27–28 weeks 2.0 cm. Some investigators may find that tracheal catheterisation is facilitated by fashioning a slight anterior curve in the catheter.

2. Draw up the surfactant (Curosurf™, Chiesi Farmaceutici, Parma, Italy) in a 3 or 5 mL syringe. The surfactant dose is 200 mg/kg (2.5 mL/kg). Draw up an additional 0.5 mL of air into the syringe, taking account of the dead volume of the instillation catheter (~0.3 mL).

3. Optional: administer atropine 20 μg/kg intravenously.

4. Disconnect standard monitors and connect the infant to the OPTIMIST oximeter (supplied to each centre)

5. The infant can be swaddled and oral sucrose administered as part of standard procedural nursing care.

#### Performing MIST

1. Position the infant as for a standard intubation procedure.

2. If possible, the laryngoscopy and tracheal cannulation should be performed with the CPAP prongs remaining *in situ*. An alternative which may improve the view of the vocal cords is to remove the CPAP prongs and apply CPAP by mask until the laryngoscopy commences.

3. Perform direct laryngoscopy using a standard laryngoscope and blade. Alternatively, use the Glidescope Cobalt AVL video laryngoscope and size 0 stat.

4. Insert the surfactant instillation catheter through the vocal cords to the desired depth, and hold it in position at the lips. The laryngoscope should then be removed.

5. Connect the surfactant syringe to the catheter hub, and instil the surfactant in 2–4 boluses over 15–30 seconds.

6. Once the surfactant is instilled, immediately remove the instillation catheter and apply CPAP by prongs or facemask.

7. If on the first attempt catheterisation of the trachea is not possible within 20–30 seconds, remove the laryngoscope, allow recovery on CPAP as required, and then attempt tracheal catheterisation once again. The maximum number of catheterisation attempts should be 3, after which the procedure should be abandoned.

#### After MIST

1. Once heart rate, SpO_2_ and respiratory effort are close to baseline values, restore the infant to their previous position, and re-establish CPAP with the same device and settings as prior to surfactant instillation.

2. Details of the procedure are recorded on a data form specifically related to the intervention. This form is then removed from the bedside by the Treatment Team, and sent to the OPTIMIST Data Management Centre in electronic format. A copy of the form should be placed in locked cabinet away from the clinical area.

3. Observations are recorded 5 minutes post-intervention, after which the OPTIMIST oximeter is disconnected and normal monitoring resumed.

4. All items that could reveal the treatment allocation to the treating clinicians should be cleared from the bedside.

Because of the novelty of the MIST technique, OPTIMIST Investigators are given the opportunity to practise the technique on an intubation mannequin during an OPTIMIST training workshop. Experience from the feasibility studies at RHH Hobart and RWH Melbourne indicates that neonatologists and neonatal fellows are highly likely to succeed in tracheal catheterisation from the outset, although two attempts at catheterisation may be required until familiarity with the technique is gained.

### Intervention – standard care (sham MIST procedure)

The following protocol is used in the standard care group:

1. Position the infant as for a standard intubation procedure. This is the only actual intervention for babies randomised to standard care. CPAP is not interrupted at any time in this group. The OPTIMIST oximeter is used and standard monitoring disconnected as for the MIST procedure.

2. Simulate the MIST procedure in terms of time taken and movement and communication within the screened area.

3. After the procedure, restore the infant to their previous position, and ensure the CPAP settings are the same as prior to the sham procedure.

4. Record the time of the sham intervention on the OPTIMIST Intervention Form, remove the form from the bedside, and send to the Data Management Centre, retaining a copy, exactly as described for the MIST procedure above.

### Management immediately after MIST

Once the MIST procedure or sham procedure is completed, the screens around the infant are removed, and care of the infant returned to the treating clinicians. For all infants, attention is drawn to the possible need to reduce the FiO_2_ so as to keep SpO_2_ in the target range. An entry is made on the drug chart to indicate the timing of the OPTIMIST study intervention. A card is placed at the bedside indicating that the infant has been enrolled in the trial, and displaying the intubation criteria. Post-intervention observations are recorded by the treating clinicians at 4 hours.

### Post-MIST investigations

A blood gas analysis (arterial or capillary) should be performed at 4 hours post-intervention, or earlier if clinically indicated.

### Post-intervention management

Other than the requirement to adhere to intubation criteria in the first week, and in some cases perform a room air trial at 36 weeks corrected gestation, management of enrolled infants after intervention is at the discretion of the clinical team. Titration of CPAP pressure according to work of breathing and oxygen requirement is encouraged. Maximum acceptable CPAP pressure is 8 cm H_2_O. NIPPV (bi-level CPAP) is allowable. Adjustment of FiO_2_ should be so as to target an SpO_2_ range appropriate for gestation and post-natal age. Prophylactic caffeine therapy would be expected in all infants [41].

#### Criteria for intubation

Infants should be intubated and ventilated if, and only if, they fulfil any of the following criteria:

1. FiO_2_ ≥ 0.45. To qualify for intubation, the FiO_2_ must be sustained at intubation level for at least 15 minutes, and all other aspects of CPAP management must have been optimised (including prong size and position, and minimisation of CPAP pressure leak).

2. Apnoea unresponsive to caffeine therapy and stimulation, which is either frequent (6 episodes in 6 hours requiring vigorous stimulation), or severe (more than one episode requiring positive pressure ventilation)

3. Persistent respiratory acidosis with pH < 7.20 and PCO_2_ > 65 mm Hg on two blood gas samples at least 30 minutes apart, or metabolic acidosis refractory to treatment

4. Need for an anaesthetic or an intervention requiring intubation

Note that these criteria apply only during the first week of life, and only for the first episode of intubation.

Once intubated, surfactant therapy can be given, at the discretion of the treating clinicians. There is no likelihood of harm if a further dose of surfactant is given less than 6 hours after surfactant administration via MIST. Thus the treating clinicians remain masked in this circumstance.

#### Assessment of BPD at 36 weeks corrected gestational age

Incidence of BPD based on oxygen requirement at 36 weeks corrected gestational age is variable within units in the Australian and New Zealand Neonatal Network (ANZNN), certainly in part due to variability in approach to oxygen therapy amongst units. Given the primacy of BPD as an outcome in the OPTIMIST-A trial, a standardised approach to its recognition has been incorporated into the trial design, based around the National Institute of Child Health and Disease consensus panel definition of “physiological BPD” [42]. On or shortly after 36 weeks 0 days corrected gestation, infants not requiring respiratory support (intubation/CPAP/HFNC ≥ 2 L/min) but receiving oxygen therapy with an FiO_2_ of less than 0.30 have a trial of room air. For infants on nasal cannula oxygen the “effective FiO_2_” is determined using the Benaron-Benitz formula [43], currently available as on online calculator (http://pub.emmes.com/study/rop/stop-js.html). Those with an FiO_2_ < 0.30 have an air trial involving stepwise FiO_2_ reductions 5 minutes apart until either room air is being administered or SpO_2_ is no longer within the target range. Based on current evidence, the minimum acceptable SpO_2_ reading for this trial is 91% [44]. A successful room air trial is defined as SpO_2_ readings ≥91% for 30 minutes in room air with nasal prongs removed [42]. Oxygen therapy can thereafter be reinstituted if deemed necessary by the treating clinicians.

Infants receiving HFNC therapy with FiO_2_ 0.21 and flow < 2 L/min also have a room air trial as above with the nasal prongs removed.

Infants requiring respiratory support, and those failing the room air trial, are deemed to have physiological BPD. BPD using the standard (clinical) definition is diagnosed if oxygen and/or respiratory support (intubation/CPAP/HFNC ≥2 L/min) is being administered for any portion of the day at 36 weeks and 0 days corrected gestational age.

Severity of BPD is categorised according to the consensus definitions [42]:

Mild BPD: need for oxygen at 28 days but not at 36 weeks corrected gestation.

Moderate BPD: need for oxygen at 28 days and continued oxygen requirement at 36 weeks (confirmed by room air trial), with FiO_2_ < 0.30.

Severe BPD: need for oxygen at 28 days, and at 36 weeks an oxygen requirement with FiO_2_ ≥ 0.30 and/or need for positive pressure support (intubation, CPAP, HFNC ≥2 L/min).

### Data collection and management

Within the OPTIMIST Investigator Team at each site, nominated personnel (e.g. Unit Data Collectors, Unit Research Nurses) collect data and enter it onto hard copy and/or electronic forms, as available. Data management is coordinated from the Clinical Epidemiology and Biostatistics Unit (CEBU) at the Murdoch Childrens Research Institute (MCRI), using a web-based database management system.

#### Data collection in hospital

Basic demographic, perinatal, and clinical data, as well as in-hospital outcomes, are collected prospectively for each patient, starting at enrolment. The data are entered on a hard copy clinical report form. Data pertaining to the MIST procedure are collected by the OPTIMIST Treatment Team, on a separate randomisation and intervention form. This form is not seen by other clinical or research staff. Once filled in, it is sent electronically to CEBU at MCRI. Information recorded includes the number of attempts required to catheterise the trachea, the total time taken, the lowest heart rate noted during the MIST procedure and time for restoration of heart rate above 100 beats per minute, the lowest SpO_2_ noted and time for restoration of SpO_2_ above 80%, and the need for and duration of positive pressure inflations by mask.

Data on heart rate, CPAP pressure, FiO_2_, and SpO_2_ prior to, and at four hours after intervention are collected, along with the results of pre- and post-intervention blood gas analysis.

#### Follow up

Each infant will have a full clinical and neurological assessment performed at 2 years by a developmental paediatrician blinded to the initial randomisation. Psychometric testing will be performed by a trained practitioner using the Bayley III Scales of Infant Development (or equivalent). Hospitalisation history in the first two years will be documented at this visit.

### Outcome variables

#### Primary outcome

Incidence of composite outcome of death or physiological BPD [42].

#### Secondary outcomes

A range of standard clinical outcomes pertaining to the first hospitalisation are being ascertained in trial participants. These are shown in Table 1. Additionally, data are being collected in the intervention group relating to the applicability and safety of the Hobart method (Table 2). For this purpose, data from the study oximeter are used alongside data recorded manually by the Treatment Team. Finally, longer term outcomes are being evaluated at two years corrected age as part of the OPTIMIST-A follow-up study. This study will have its own protocol and funding stream. Selected outcomes from this study are shown in Table 3.

**Table 1:** Clinical outcomes during first hospitalisation

Physiological BPD [42]	Duration of intubation (all episodes)
Clinical BPD (oxygen or positive pressure support at 36 weeks corrected gestation) [45]	Duration of CPAP/NIPPV (all episodes)
Mild/moderate/severe BPD [45]	Duration of intubation and CPAP
Death	Duration of HFNC, minimum flow rate 2 L/min
Death or BPD (clinical definition)	Duration of respiratory support
Intraventricular haemorrhage (IVH) (all grades)	Duration of oxygen therapy
IVH grades III and IV [46]	Requirement for oxygen at home
Cystic periventricular leukomalacia	Length of stay in intensive care
Retinopathy of prematurity (ROP) > stage II	Length of hospital stay
Major morbidity (any of IVH grade III or IV, periventricular leukomalacia, ROP > stage II, physiological BPD) [47]	Total hospital billings
Death or major morbidity	Calculated cost of hospitalisation
NEC (Modified Bell stage 2 or greater) [48]	Pneumothorax requiring drainage
NEC or spontaneous intestinal perforation requiring surgery	Pulmonary haemorrhage
Requirement for intubation < 72 h	Patent ductus arteriosus (PDA) requiring anti-prostaglandin therapy
Requirement for intubation at any time	PDA requiring ligation
Need for additional surfactant therapy	Late onset sepsis (positive bacterial or fungal culture from a normally sterile site)
Overall number of surfactant doses (including that given by MIST)	Time to regain birth weight

**Table 2:** Applicability and safety outcomes in infants randomised to receive surfactant via the Hobart method

Incidence of successful surfactant administration via MIST	Duration of hypoxaemia (SpO_2_ < 80%)
Number of catheterisation attempts	Requirement for, and duration of, positive pressure ventilation by mask
Duration of bradycardia (heart rate < 100 beats per minute)	Incidence of apparent discomfort

**Table 3:** Selected outcomes from the OPTIMIST-A follow-up study

Number of hospitalisations in the first 2 years	Major disability at 2 years
Number of hospitalisations with respiratory illness in the first 2 years	Death or major disability at 2 years

### Statistical analysis and reporting

#### Statistical analysis

Data handling, verification and analysis for the OPTIMIST-A trial are being performed by CEBU at MCRI. Statistical analysis will follow standard methods for randomised trials and the primary analysis will be by intention to treat. For dichotomous outcomes, including the primary outcome in OPTIMIST-A, proportions will be compared using the odds ratio with 95% CI, obtained from a logistic regression analysis with adjustment for the strata (defined by centre and gestational age category) used in the randomisation. Continuous outcomes will be compared using differences between mean values, estimated from normal linear regression models with the same stratification adjustments. Secondary analyses will use expanded regression models to explore potential confounding effects of chance imbalances between arms in birth weight, gender, antenatal steroids, or mode of delivery. In further secondary analysis, we will explore evidence for heterogeneity of effects between the two gestational age strata in the trial, using interaction tests and subgroup analyses.

#### Data reporting and manuscript preparation

A clinical study report will be generated from the Data Management Centre. This document will, after approval by the Trial Steering Committee, form the basis of conference presentations and manuscripts for publication. In all cases data reporting will adhere to the CONSORT guidelines. Responsibility for manuscript preparation will rest with the Trial Steering Committee. Authorship will be in the form of: Author A, Author B, Author C,… for the OPTIMIST-A Investigators.

### Sample size

In the RHH-RWH CPAP study, amongst infants of 25–28 weeks gestation the proportion positive for the outcome of death or BPD was 53% in those failing CPAP and 38% in those reaching the OPTIMIST-A enrolment threshold in the first 2 hours. A reduction by one-third in the proportion of infants with this outcome (i.e. from 38% to 25%) would be a major advance in care for this patient group, relieving the burden at both individual and NICU levels. Detection of a reduction of this magnitude with 90% power and α = 0.05 (two-sided) would require 297 subjects per arm [49]. An allowance has been made for withdrawal of 2% of subjects post-recruitment. The number of subjects to be randomised in each arm is thus 303, for an overall total of 606.

### Trial plan

Australasian neonatal Units and selected international centres, including those in the Vermont-Oxford network, are being invited to join the trial, and local information sessions held in interested centres as required. The Trial Coordinating Centre Team assists Units joining the study with ethics submissions and organisational matters.

At the time of study commencement at each site, a trial workshop is being conducted by a team from the Trial Coordinating Centre. These workshops consist of 1) a formal outline of the trial, 2) a hands-on demonstration of the MIST technique using an intubation mannequin, 3) a bedside simulation of the MIST procedure and of the sham intervention by an OPTIMIST Treatment Team, and 4) in-depth discussion of the practicalities of screening, randomisation and data collection.

A full complement of participating centres is expected for the OPTIMIST-A trial by mid-2015. Recruitment will thereafter proceed at full rate until completion, which is estimated to be completed at the end of 2017.

### Data and safety monitoring committee

An independent Data and Safety Monitoring Committee (DSMC) has been established for the OPTIMIST-A trial. The terms of reference for this committee includes performance of interim data analysis, periodic examination of emerging external evidence in relation to MIST, and monitoring of adverse events, compliance with the trial protocol, and progress of recruitment. The DSMC has developed their own charter for the conduct of these and other activities [50].

#### Interim analyses

The Trial Steering Committee expects that there will be a maximum of three interim analyses for the OPTIMIST-A trial. Relevant event rates in enrolled infants will be compared to the background rates in data from the RHH-RWH preterm CPAP study, and recommendations for change in sample size made if a substantial disparity is noted. The odds ratios for major outcomes will be examined in the two randomisation groups. For this comparison the statistical approach will be conservative, with a recommendation to cease the trial on efficacy grounds only to be made in the presence of very strong interim evidence. Later analyses will also include consideration of whether there is an unequivocal lack of efficacy. At each meeting of the DSMC the ethical position in relation to further randomisation will be considered based on results of other randomised controlled trials comparing MIST with standard care (continuation of CPAP).

#### Adverse events

Serious adverse events (SAEs), including those leading to death, prolonged hospitalisation or persistent disability, are relatively common in preterm infants 25–28 weeks gestation. Serious adverse events which are in the opinion of the local investigator unexpected, are to be reported within 5 working days to the coordinating centre. The SAE will then be reported to the local Ethics Committee, the Tasmania Health and Medical Ethics Committee, the Trial Steering Committee, the DSMC, and, as appropriate, the Therapeutic Goods Administration and/or other federal regulatory bodies. The data pertaining to the SAE will be examined by the DSMC, and any recommendations made will be disseminated to local investigators.

### Funding

Funding has been obtained for commencement of the OPTIMIST-A trial from the RHH Research Foundation (for 2011 and 2012), and from the Australian NH&MRC for the years 2013–2017. Chiesi Farmaceutici, Parma, Italy has agreed to supply the surfactant at significantly reduced cost. The trial sponsor is the Menzies Research Institute Tasmania, and the trial insurer is Unimutual Limited.

## Discussion

This trial is the next logical step in refining the care provided to preterm infants 25–28 weeks gestation. It will be by far the largest randomised controlled trial investigating MIST conducted to date, and in its own right will have sufficient power to give definitive information about the place of this therapy. The concept of MIST is exciting because of the possibility that giving surfactant in this way to preterm infants may improve respiratory outcomes including duration of respiratory support and risk of BPD. A reduction in the incidence of BPD in the 25–28 week infants would represent a significant advance for this group, in which the risk of BPD remains high, and leads in many cases to chronic respiratory ill health in infancy.

## Endnote

^a^OPTIMIST-A: The first of a pair of clinical trials investigating MIST in preterm infants at different gestation ranges (25–28 and 29–32 weeks). The acronym is derived from C***o***llaborative ***P***aired ***T***rials ***I***nvestigating ***M***inimally-***I***nvasive ***S***urfactant ***T***herapy.

## Abbreviations

ANZNN: Australian and New Zealand neonatal network; BPD: Bronchopulmonary dysplasia; CEBU: Clinical epidemiology and biostatistics unit, Murdoch childrens research institute; CPAP: Continuous positive airway pressure; DSMC: Data and safety monitoring committee; FiO_2_: Fraction of inspired oxygen; HFNC: High flow nasal cannulae; INSURE: Intubation, Surfactant, Extubation; MCRI: Murdoch childrens research institute; MIST: minimally-invasive surfactant therapy; NEC: Necrotizing enterocolitis; NHMRC: National health and medical research council (Australia); NICU: Neonatal intensive care unit; OPTIMIST-A: The first of a pair of clinical trials investigating MIST in preterm infants at different gestation ranges (25–28 and 29–32 weeks). The acronym is derived from C***o***llaborative ***P***aired ***T***rials ***I***nvestigating ***M***inimally-***I***nvasive ***S***urfactant ***T***herapy; PDA: Patent ductus arteriosus; RDS: Respiratory distress syndrome; RHH: Royal Hobart hospital, Hobart; RWH: Royal women’s hospital, Melbourne; SAE: Serious adverse event; SpO_2_: Oxygen saturation measured by pulse oximetry.

## Competing interests

Surfactant for the intervention in the OPTIMIST-A trial is being provided at reduced cost by Chiesi Farmaceutici. The authors declare no other financial or non-financial competing interests.

## Authors’ contributions

PAD: Conceived the study, made intellectual contribution to the study protocol, wrote the first draft of the manuscript. OK/AGdeP/JBC/FO/RFS/PGD: Made intellectual contribution to the study protocol, read and edited the manuscript. All authors read and approved the final manuscript. The authors of this manuscript constitute the membership of the OPTIMIST-A Trial Steering Committee.

## Authors’ information

**PAD** MBBS FRACP MD is a Neonatologist and Director of the Neonatal & Paediatric Intensive Care Unit, Royal Hobart Hospital, Hobart, and an Honorary Research Fellow at the Menzies Research Institute Tasmania.

**COFK** FRACP DMedSci is a Neonatologist at the Royal Women’s Hospital, Melbourne.

**AGdeP** FRACP MD is a Neonatologist at the Neonatal & Paediatric Intensive Care Unit, Royal Hobart Hospital, Hobart.

**JBC** BSc(Hons) PhD is Head of the Clinical Epidemiology and Biostatistics Unit, Murdoch Childrens Research Institute, University of Melbourne, Melbourne.

**FO** BSc MSc is a statistician at the Clinical Epidemiology and Biostatistics Unit, Murdoch Childrens Research Institute, Melbourne.

**RFS** MD PhD is H. Wallace Professor of Neonatology at the University of Vermont.

**PGD** FRACP MD is Professor of Neonatal Medicine at the Royal Women’s Hospital, Melbourne.

## Pre-publication history

The pre-publication history for this paper can be accessed here:

http://www.biomedcentral.com/1471-2431/14/213/prepub
